# Supplementary material for: Investigation of Biomaterial Ink Viscosity Properties and Optimization of the Printing Process Based on Pattern Path Planning
Source: Bioengineering (Basel). 2023 Nov 26;10(12):1358. doi: 10.3390/bioengineering10121358 (PMC10740413; doi:10.3390/bioengineering10121358)
Supplement: Supplementary file 1 [file bioengineering-10-01358-s001.zip › bioengineering-2693265-supplementary.pdf]

Supplementary Information

The model of computational fluid dynamics was built on the basis of the actual dimensions of the 25G needle tip, which retained the important features of the tip, as shown in Fig. S1.

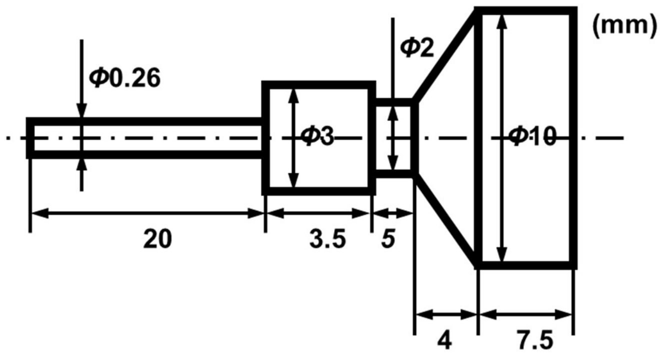

Fig. S1 Simulation model of the nozzle.

The relationship between the viscosity property of biomaterial inks and the concentration of compositions was explored by response surface methodology (RSM), with 17 sets of experimental points designed and randomized by Box-Behnken method. Rheological experiments were carried out to obtain the power-law index (n) and the consistency coefficient (K) of the biomaterial inks formulated at each experimental point, as shown in Table S1. Afterwards, the results were transferred to RSM for data analysis and the model was validated by ANOVA, as shown in Tables S2 and S3.

Table S1 RSM experimental design and results for the rheological properties of biomaterial inks

Order	Gel (wt%)	SA (wt%)	MC (wt%)	n	K (Pa·s)
1	6	5	3	0.61	42.06
2	8	4	3	0.59	48.80
3	8	3	2	0.66	19.17
4	8	4	3	0.61	41.95
5	10	4	2	0.50	85.08
6	6	3	3	0.67	15.91
7	8	3	4	0.55	60.41
8	10	3	3	0.49	78.82
9	8	5	2	0.57	59.98
10	10	5	3	0.36	281.20
11	8	4	3	0.65	37.38
12	8	4	3	0.58	44.54
13	6	4	2	0.67	18.84
14	8	5	4	0.44	192.12
15	6	4	4	0.63	38.30
16	8	4	3	0.58	52.30
17	10	4	4	0.36	276.61

Table S2 ANOVA for the power-law index

Source	df	Adj SS	Adj MS	F-value	P-value
Model	7	0.1500	0.0214	37.98	<0.0001
Gel	1	0.0923	0.0923	163.61	<0.0001
SA	1	0.0180	0.0180	31.87	0.0003
MC	1	0.0225	0.0225	39.85	0.0001
Gel*Gel	1	0.0083	0.0083	14.62	0.0041
SA*SA	1	0.0028	0.0028	5	0.0523
MC*MC	1	0.0019	0.0019	3.39	0.0985
Gel*MC	1	0.0029	0.0029	5.15	0.0494
Lack of Fit	5	0.0015	0.0003	0.35	0.8624
Pure Error	4	0.0035	0.0009		
Cor Total	16	0.1551			

R-Squared: 96.73%, adjusted R-Squared: 94.18%, predicted R-Squared: 91.02%.

Table S3 ANOVA for the consistency coefficient

Source	df	Adj SS	Adj MS	F-value	P-value
Model	9	112735	12526.1	124.37	<0.0001
Gel	1	45993	45992.8	456.65	<0.0001
SA	1	20105	20105	199.62	<0.0001
MC	1	18468	18468.3	183.37	<0.0001
Gel*Gel	1	6956	6956.1	69.06	0.0001
SA*SA	1	1497	1497.2	14.87	0.0062
MC*MC	1	1530	1530.4	15.19	0.0059
Gel*SA	1	7764	7764.2	77.09	0.0001
Gel*MC	1	7402	7402	73.49	0.0001
SA*MC	1	2066	2065.5	20.51	0.0027
Lack of Fit	3	570	189.9	5.62	0.0644
Pure Error	4	135	33.8		
Cor Total	16	113440			

R-Squared: 99.38%, adjusted R-Squared: 98.58%, predicted R-Squared: 91.78%.

The neural network model was validated by randomly generating 50 sets of experimental points and calculating the flow rate at the nozzle based on CFD and neural network, respectively, as shown in Table S4.

Table S4 Simulation validation of the neural network model

n	K (Pa·s)	P (kPa)	Q_{CFD} (μL)	Q_{BP} (μL)
0.554	67.989	338.927	0.222	0.228
0.462	44.075	214.042	0.553	0.565
0.676	83.042	206.341	0.034	0.042
0.686	57.954	119.478	0.025	0.020
0.573	41.565	196.669	0.174	0.173
0.572	94.510	237.218	0.058	0.055
0.534	88.835	253.741	0.093	0.102
0.675	59.514	168.655	0.042	0.039
0.542	66.023	160.231	0.065	0.064
0.478	62.834	346.395	0.595	0.574
0.645	28.697	61.322	0.031	0.044
0.547	37.112	315.550	0.628	0.629
0.510	52.383	323.986	0.512	0.509
0.551	30.744	288.855	3.649	3.668
0.474	85.988	79.614	0.014	0.017
0.483	27.529	128.561	0.397	0.417
0.686	30.333	150.607	0.090	0.095
0.689	25.364	253.918	0.245	0.242
0.594	30.490	90.966	0.068	0.069
0.465	49.213	266.368	0.677	0.671
0.509	37.999	82.029	0.065	0.056
0.538	93.104	246.127	0.078	0.083
0.655	48.719	198.252	0.079	0.082
0.454	26.633	283.716	0.727	0.731
0.461	91.439	264.511	0.180	0.190
0.492	98.177	321.116	0.172	0.178
0.612	49.498	317.277	0.215	0.212
0.633	20.001	150.249	0.237	0.236
0.612	33.226	259.624	0.298	0.300
0.563	46.785	109.343	0.054	0.053
0.587	63.541	59.162	0.010	0.020
0.524	33.599	273.222	0.749	0.742
0.636	64.256	200.007	0.058	0.055
0.497	74.009	193.977	0.105	0.112
0.622	29.957	321.417	0.459	0.470
0.496	20.568	232.960	2.104	2.082
0.542	36.701	235.300	0.394	0.390
0.606	38.690	307.833	0.321	0.321
0.645	48.175	291.647	0.155	0.152
0.470	55.707	223.016	0.330	0.329

0.682	17.696	104.877	0.119	0.109
0.644	33.623	121.980	0.071	0.075
0.572	82.091	315.954	0.122	0.125
0.559	12.630	58.602	0.188	0.184
0.562	93.597	196.970	0.045	0.038
0.527	75.730	100.378	0.023	0.022
0.577	53.975	343.604	0.282	0.282
0.578	62.067	263.808	0.139	0.147
0.654	31.356	200.141	0.158	0.160
0.649	51.296	191.327	0.072	0.073

Mean Absolute Error: 0.60%, Root Mean Square Error: 0.81%.

The Φ15 × 5 mm samples were prepared by varying the concentrations of gelatin (7wt%, 8wt%, 9wt%) or methylcellulose (2wt%, 3wt%, 4wt%), respectively, and the compression modulus was measured after crosslinking in 5 wt% $CaCl_2$ for 30 min.

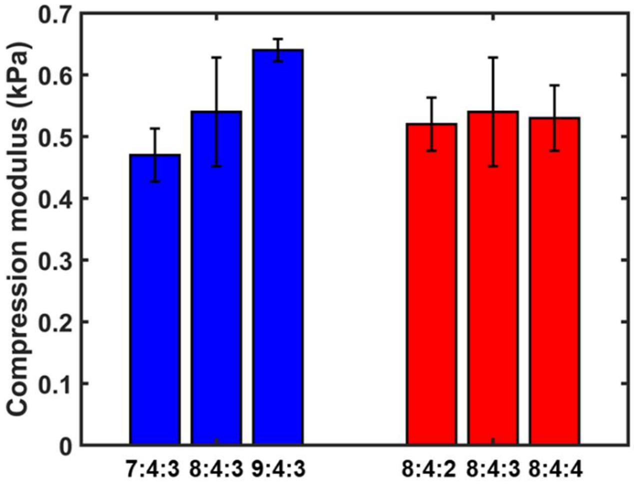

Fig. S2 Compression modulus of biomaterial inks with different concentrations of gelatin or methylcellulose

Four layers of grid-filled quadrilateral prisms were printed with 8:3:3, 8:4:3, and 8:5:3 biomaterial inks, respectively. Then the widths and shrinkage ratio of 8 filaments before and after crosslinking were measured and calculated.

Table S5 Widths and shrinkage ratio of 8 filaments before and after crosslinking*

Biomaterial inks	Width of filament (Uncrosslinked) (mm)	Width of filament (Crosslinked) (mm)	Shrinkage ratio
8:3:3	0.29 ± 0.062	0.32 ± 0.027	-8.7%
8:4:3	0.38 ± 0.045	0.33 ± 0.023	12.3%
8:5:3	0.48 ± 0.030	0.44 ± 0.027	9.8%

* The values were averaged from 8 filaments taken from each sample.

A solid and a grid-filled quadrilateral prism with dimension of $10 \times 10 \times 3\ mm^3$ were printed with 8:4:3 biomaterial ink. The blue circle is the starting points for each layer of printing, and the green dotted line is the outline of the first layer, all of which maintained high fidelity.

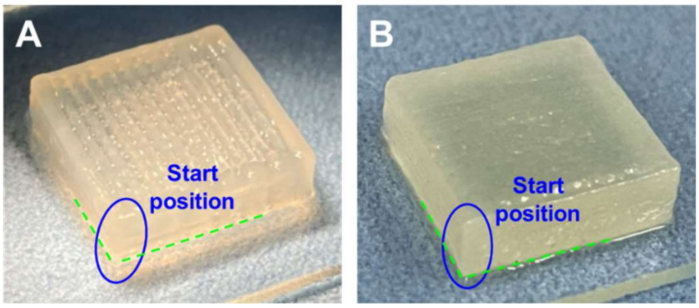

Fig. S3 Prints with dimension of $10 \times 10 \times 3\ mm^3$ after crosslinking. (A) The solid quadrilateral prism after crosslinking. (B) The grid-filled quadrilateral prism after crosslinking.
